# Distribution modeling and lineage diversity of the chytrid fungus *Batrachochytrium dendrobatidis* (*Bd*) in a central African amphibian hotspot

**DOI:** 10.1371/journal.pone.0199288

**Published:** 2018-06-20

**Authors:** Courtney A. Miller, Geraud Canis Tasse Taboue, Mary M. P. Ekane, Matthew Robak, Paul R. Sesink Clee, Corinne Richards-Zawacki, Eric B. Fokam, Nkwatoh Athanasius Fuashi, Nicola M. Anthony

**Affiliations:** 1 Department of Biological Sciences, University of New Orleans, New Orleans, Louisiana, United States of America; 2 Department of Zoology and Animal Physiology, University of Buea, Buea, Cameroon; 3 Institute of Geological and Mining Research, Yaoundé, Cameroon; 4 Department of Ecology and Evolutionary Biology, Tulane University, New Orleans, Louisiana, United States of America; 5 Department of Biology, Drexel University, Philadelphia, Pennsylvania, United States of America; 6 Department of Biological Sciences, University of Pittsburgh, Pittsburgh, Pennsylvania, United States of America; Universitat Trier, GERMANY

## Abstract

The amphibian disease chytridiomycosis in amphibians is caused by the chytrid fungus *Batrachochytrium dendrobatidis* (*Bd*) and has resulted in dramatic declines and extinctions of amphibian populations worldwide. A hypervirulent, globally-dispersed pandemic lineage (*Bd*-GPL) is thought to be largely responsible for population declines and extinctions, although numerous endemic lineages have also been found. Recent reports of amphibian declines have been linked to the emergence of *Bd* in Cameroon, a major hotspot of African amphibian diversity. However, it is not known whether *Bd*-GPL or other lineages have been found in this region. This study therefore aims to examine *Bd* lineage diversity in the region and predict the distribution of this pathogen under current and future climate conditions using data from this study and from historical records. Almost 15% (52/360) of individuals tested positive for *Bd* using a standard quantitative PCR diagnostic. Infected amphibians were found at all eight sites sampled in this study. Species distribution models generated in BIOMOD2 indicate that areas with highest predicted environmental suitability occur in the Cameroon highlands and several protected areas throughout the country. These areas of high environmental suitability for *Bd* are projected to shift or decrease in size under future climate change. However, montane regions with high amphibian diversity are predicted to remain highly suitable. Phylogenetic analysis of the ITS sequences obtained from a set of positive *Bd* samples indicate that most fall within the *Bd*-GPL lineage while the remainder group with isolates from either Brazil or South Korea. Although more in depth phylogenetic analyses are needed, identification of *Bd*-GPL lineages in areas of high amphibian diversity emphasizes the need to continue to monitor for *Bd* and develop appropriate conservation strategies to prevent its further spread.

## Introduction

The infectious disease chytridiomycosis is a leading cause of global amphibian population declines. Clinical symptoms of the disease most commonly include excessive shedding of the skin, hyperkeratosis, and skin redness or discoloration. In general, the disease is diagnosed by the presence of maturing zoosporangia of the amphibian chytrid fungus, *Batrachochytrium dendrobatidis* (*Bd*) which infects the keratin-containing layers of amphibian skin [1]. According to a recent global assessment, *Bd* has been detected in over 500 amphibian species [2] and is established on every continent where amphibians are found. In Africa, *Bd* has been documented in South Africa [3], the Albertine Rift in the Democratic Republic of Congo [4] and Uganda [5], Ethiopia [6], Kenya [7], Tanzania [8], Malawi [9], Morocco [10], and recently Mozambique [11]. Across the rainforest belt of central equatorial Africa, *Bd* presence has been reported in Gabon [12], Nigeria [13,14], the island of Sao Tomé [15], and Cameroon [8,16,17]. Most of these reports from Africa consist of presence/absence assessments and estimations of *Bd* prevalence, thus there is little known about lineage diversity within *Bd* in this region.

Within the Central African region, the Cameroon highlands and flanking lowland forests represent a globally-important biodiversity hotspot [18,19]. With approximately 200 anuran species described [20], Cameroon has the third highest amphibian species richness in Africa, only exceeded by Madagascar and the Democratic Republic of Congo [21]. It also has the highest percentage of endemic amphibians in mainland Africa, 88% of which occur in the highlands [22]. Studies reporting *Bd* in the region have not found evidence for the effects of chytridiomycosis. However, recent analyses of frogs in montane areas of Cameroon reported that community-wide amphibian declines were correlated with *Bd* prevalence rather than habitat loss or land use change [23]. Increasing our understanding of *Bd* presence and the likelihood of this pathogen spreading under future climate change throughout this amphibian hotspot is essential for proactive conservation planning.

Species distribution models (SDMs) have proven to be useful tools for predicting *Bd* distribution [24–26] as several environmental factors are thought to limit its range. In general, *Bd* presence is associated with relatively cool to moderate temperatures of between 17 and 25°C [27] although it can survive temperatures between 4 and 28°C [28]. *Bd* infection prevalence has been shown to increase with cooler conditions and decrease with greater temperature [26]. Population declines and extinctions in amphibian hosts are also more likely in cooler, wetter, more thermally stable regions [29] and deaths from chytridiomycosis are common in high-altitude [30] and tropical montane regions [24,25]. *Bd* prevalence has also been shown to vary significantly by season [31] with the highest prevalence reported for cooler months [31–33]. *Bd* reproduction requires moist environments and several measures of precipitation have been demonstrated as significant predictors of *Bd* suitability [3,26,34].

Phylogenetic analyses indicate far greater endemic diversity in *Bd* than has been previously recognized [35,36]. To date, diverged lineages include BdCAPE (South Africa), BdASIA-1 (which includes Korea and a single BdCH isolate from Switzerland), BdASIA-2/BdBRAZIL (Korea and Brazil) and the global panzootic lineage, *Bd*-GPL [36]. *Bd*-GPL is the most globally widespread lineage and primarily associated with amphibian die-offs, leading some researchers to suggest that it has replaced rarer, less-virulent enzootic lineages [37]. Korea has been recently confirmed as the geographic origin of *Bd* and its distribution linked to the global commercial trade of amphibian [36]. Several *Bd* samples from Gabon and the islands Bioko, São Tomé, and Príncipe were recently characterized as *Bd*-GPL [38]. However, our knowledge of the distribution of *Bd*-GPL elsewhere in the rainforests of Equatorial Africa is poor.

To better understand predicted *Bd* distributions in a known biodiversity hotspot in the central African country of Cameroon, we combined data from *Bd* surveys carried out at eight sites in Cameroon with other available regional records to model environmental suitability for *Bd* under current environmental conditions and future climate change scenarios. We also sequenced the ITS1-5.8S-ITS2 region of the *Bd* genome from a subset of samples to estimate phylogenetic relationships of Cameroonian samples to determine if *Bd* in Cameroon identify as endemic lineage(s) or are characteristic of the globally dispersed pandemic lineage (*Bd-*GPL).

## Materials and methods

### Ethics statement

Protocols for this study were approved by the University of New Orleans Institutional Animal Care and Use Committee (IACUC) under protocol number 12–008. Field work was approved by the Cameroon Ministry of Forestry and Wildlife (MINFOF), under research permit number 0984/PRS/MINFOF/SG/DF/SDVEF/SC. All researchers involved in field work were issued research permits to enter the national parks and wildlife reserves by the Cameroonian Ministry of Scientific Research and Innovation (MINRESI).

### Field surveys

**Fig 1:**
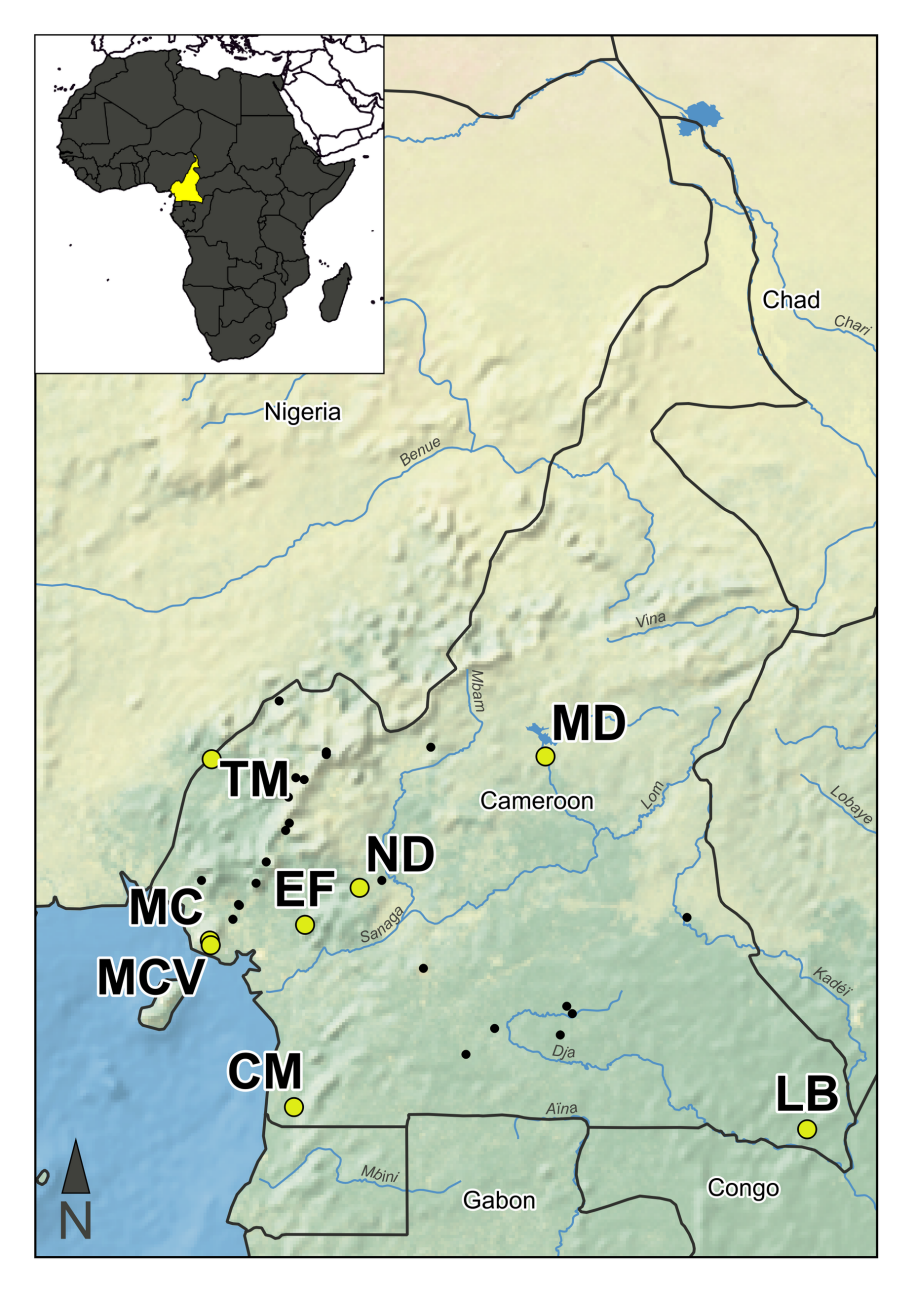
Map of the study region. Locations of the study sites, Campo Ma’an National Park (CM), Ebo Forest (EF), Lobéké National Park (LB), Mbam Djerem National Park (MD), Mount Cameroon village (MCV), Mount Cameroon (MC), Ndikiniméki (ND), and Takamanda (TM) are marked with yellow circles. Previously published *Bd* sample sites are marked with black circles. The sites sampled in this study have all been previously sampled with the exception of Mbam Djerem National Park and Takamanda. This figure was created with Natural Earth vector and raster map data (naturalearthdata.com).

A total of 360 amphibians from eight sites (average of 46 specimens per site) across Cameroon were swabbed for *Bd* during nocturnal surveys carried out in May–July in 2014 and March-April in 2015 (Fig 1, S1 Table). Post-metamorphic terrestrial and arboreal frogs were collected by hand using sterile nitrile gloves. To avoid potential cross-contamination, frogs were placed in individual plastic re-sealable bags and gloves were changed between individuals. Frogs were swabbed for *Bd* with sterile rayon swabs (Medical Wire & Equipment Co., Corsham Wilshire UK) by stroking the ventral surfaces of their thighs, abdomen, and foot webbing approximately five times each [39]. Swabs were placed in Eppendorf tubes containing 95% ethanol and stored at room temperature prior to their transfer to the laboratory. Frogs were identified to genus or species level and then released at their point of capture.

### Molecular diagnostics for *Bd*

Genomic DNA was extracted from swab samples using a Qiagen DNAeasy Blood and Tissue kit, following the manufacturer’s protocol, with a final elution volume of 200μl. A quantitative, real-time TaqMan quantitative PCR (qPCR) assay (Applied Biosystems, CA) was used to diagnose the presence and quantity of *Bd* from the extracted DNA following Boyle et al. [40]. Prior to amplification, each sample was diluted 1:10 with double-distilled water and 0.7μl of bovine serum albumin was added to each reaction. An internal positive control (VIC^TM^ IPC, Applied Biosystems) was added to each qPCR reaction to confirm that PCR inhibition was not affecting our results. A negative control and a series of plasmid dilution standards, with concentrations ranging from 2.1 x 10^0^–2.1 x 10^6^ copies per μL (Pisces Molecular) were included in each qPCR run. While qPCRs are extremely sensitive, samples collected from swabs can contain relatively low levels of DNA, hence samples were run up to three times to screen for *Bd*. Samples were considered positive if the qPCR assay detected > 0.9 copies of the *Bd* target region in a 5 μL sample of extracted DNA. Whole swab infection intensity for individual amphibians was quantified as plasmid equivalents (PE) [41–43], or DNA copies, by multiplying qPCR results by 40 to account for the portion of the 200μl extract that was not included in the qPCR, then multiplying by the dilution factor of 10. This gives an estimate of the number of DNA copies on the whole swab. Upper and lower confidence intervals (95%) for *Bd* prevalence were calculated using the R package PropCIs [44].

### Species distribution modeling

Current and projected future SDMs of *Bd* in Cameroon were created using the BIOMOD2 package [45] implemented in the statistical program R, version 3.3.0 [46]. BIOMOD2 is a framework for ensemble forecasting of species distributions using species occurrence data and environmental predictor variables. It can incorporate up to 11 different modeling algorithms and generate ensemble projections that are weighted by model performance. Eight modeling algorithms in BIOMOD2 were used to assess environmental suitability for *Bd*: generalized linear models (GLM, [47]), generalized additive models (GAM, [48]), generalized boosting models (GBM, [49]), classification tree analysis (CTA, [50]), flexible discriminant analysis (FDA, [51]), multivariate adaptive regression splines (MARS, [52]), random forest (RF, [53]), and the maximum entropy approach modeled in Maxent [54]. Model calibration was performed using random sampling (70%) of the data as implemented in BIOMOD2. Model evaluation was carried out with the true skill statistic (TSS) [55], using the remaining 30% of the data over the 10 model replicate runs. Only models with a TSS > 0.6 were incorporated into the final ensemble model. True skill statistic scores are not affected by prevalence and range from -1 to 1, with 0 indicating no predictive ability and 1 indicating a perfect ability. Species occurrence data were comprised of *Bd* presence and absence points from the current study as well as from the primary literature [6,16,56]. Supplementary *Bd* occurrence records (187 presence points and 739 absence points) were collected from Cameroon and neighboring regions (southern Nigeria and Gabon). Only sources that used qPCR as the detection method and involved specimens collected in the field were included (S2 Table).

Nineteen bioclimatic variables [57] and elevation [58] were considered as predictors in species distribution models. Highly correlated variables (R > 0.8) were eliminated based on Pearson’s correlation coefficients using the R package virtualspecies [59]. After comparing model performance for sets of uncorrelated variables, the following predictive environmental variables were selected to model *Bd* distributions under present conditions: annual temperature, isothermality (temperature evenness), minimum temperature of the coldest month, mean temperature of the coldest quarter, annual precipitation, and elevation. Future projections of the bioclimatic variables [60] were created by aggregating 20 available global climate models for the four primary potential climate change scenarios, termed representative concentration pathways (RCP) 2.6, 4.5, 6.0, and 8.5, for years 2030, 2050, and 2080 based on the Intergovernmental Panel on Climate Change (IPCC) 5^th^ assessment report at a spatial resolution of 30 arc-seconds (approximately 1km^2^) [61,62].

### Phylogenetic analysis of *Bd* sequences

A 300bp region encompassing the 5.8S rRNA gene and portions of both flanking internal transcribed spacers (ITS1, ITS2) was amplified from 14 *Bd* positive samples using ITS primers *Bd*1a (5’-CAGTGTGCCATATGTCACG-3’) and *Bd*2a (5’-CATGGTTCATATCTGTCCAG- 3’) [63,64]. Polymerase chain reaction (PCR) assays were conducted with 2 μL of each template DNA in a total reaction volume of 12 μL. The PCR reaction mix contained 1x PCR Buffer (Invitrogen, CA), 1.5 mM MgCl_2_, 0.2 mM of each dNTP, 0.5 mM of each primer, and 1.25 units of Taq DNA polymerase (Invitrogen, CA).

PCR amplification was performed using an initial denaturation at 95°C for 5 min followed by 44 cycles of denaturation at 93°C for 45 s, an annealing step at 60°C for 45 s, an extension step at 72°C for 1 min, and a final extension at 72°C for 10 min. Excess primers and unincorporated nucleotides were removed with ExoSAP-IT (Affymetrix) and PCR products were Sanger sequenced in both the forward and reverse directions (Hitachi 3130*xl*, Applied Biosystems). Sequences were trimmed in Geneious software [65] and aligned with an additional 189 sequences from previous studies (S3 Table) using the MUSCLE alignment implemented in MEGA7 [66]. Another genus of Chytridiomycota, *Kappamyces laurelensis* (ITA2582), was used as the outgroup [64]. Bayesian phylogenetic inference was performed with MrBayes v3.2.6 [67] under a TIM1+G model of evolution determined as the best model using jModelTest v.2.1.1 [68,69]. Bayesian analyses were run with four MCMC chains for 10 million generations and sampled every 1000 generations. The phylogenetic tree and posterior probabilities for individual nodes was visualized using FigTree v1.4.3 [70].

## Results

### Spatial distribution, prevalence and infection intensity of *Bd* and its host associations

In total, 52 frogs representing 5 families, 11 genera, and 23 species tested positive for *Bd*, with an average prevalence of 14.4% (Table 1). *Bd* was found at all eight sites sampled across Cameroon. The Mt. Cameroon Village site in the highlands had the highest prevalence (33.3%, N = 15), whereas Ebo Forest in central Cameroon had the lowest (9.2%, N = 130). Average infection intensity was 7023 ±24109.1 PE (N = 52) (S4 Table). The sites with the highest infection intensity were Ndikiniméki (27567 ± 53910.6 PE, N = 4) and Ebo Forest (17943.6 ± 38734.5 PE, N = 12) (Table 1). The individuals with the highest infection intensities were *Alexteroon obstetricians* (PE = 117508), *Xenopus andrei* (PE = 80512) from Ebo Forest, and *Arthroleptis* aff. *poecilonotus* (PE = 108432) from Ndikiniméki. The two genera with the highest prevalence were *Xenopus* (42.9%, N = 7) and *Arthroleptis* (30.8%, N = 39) (S5 Table). However, none of the amphibians collected exhibited clinical signs of chytridiomycosis in the form of skin or behavioral abnormalities.

**Table 1:** Prevalence and infection intensity of *Bd* from eight sites in Cameroon. Prevalence (with 95% confidence intervals) and infection intensity, mean and standard deviation (SD), is reported for Campo Ma’an (CM), Ebo Forest (EF), Lobéké National Park (LB), Mbam Djerem National Park (MD), Mount Cameroon (MC), Mount Cameroon Village (MCV), Ndikiniméki (ND), and Takamanda (TM).

								Infection Intensity (PE)	
Site	Lat	Long	Elevation (m)	No. species	No. specimens	No. Bd positive	% Prevalence (95% CI)	Mean	SD
CM	2.44	10.20	93–465	25	64	10	15.63 (8.71–26.43)	15.3	4.06
EF	4.55	10.29	94–993	25	130	12	9.23 (5.36–15.44)	448.6	968.36
LB	2.38	15.38	152–571	20	48	12	25 (14.92–38.78)	38.5	39.48
MD	5.32	13.60	603–887	3	4	1	25 (4.56–69.94)	17.7	-
MC	4.13	9.12	1935–2075	1	17	4	23.53 (9.56–47.26)	30	15.59
MCV	4.14	9.20	967–1327	6	15	5	33.33 (15.18–58.29)	23.2	9.61
ND	4.84	10.87	728–795	12	41	4	9.76 (3.59–21.16)	689.2	1347.77
TM	6.13	9.22	120–126	10	41	4	9.76 (3.59–21.16)	30.4	28.31
Total					360	52	14.44 (11.19–18.45)	175.58	602.73

### Species distribution modeling

**Fig 2:**
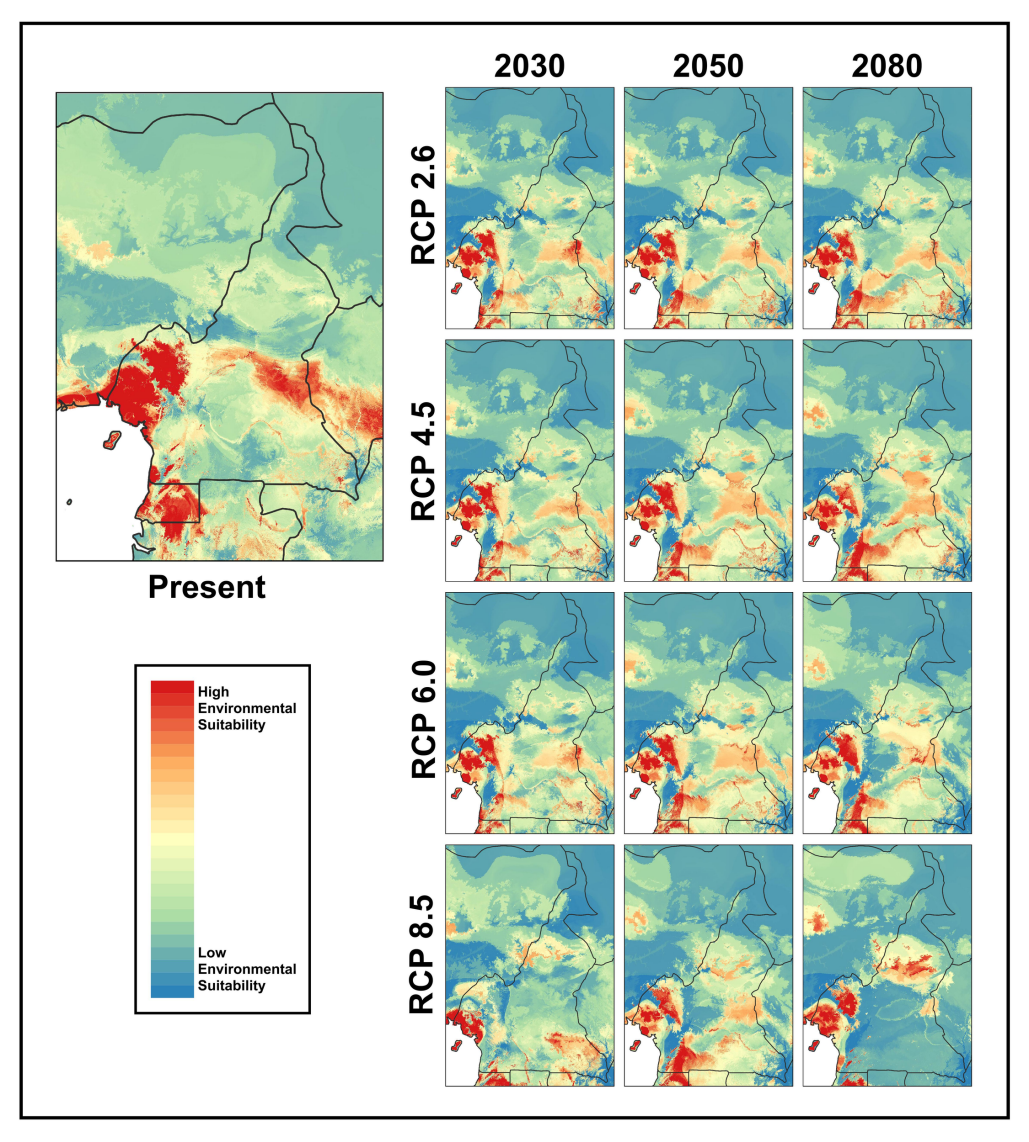
Environmental suitability modeled in BIOMOD2 for *Bd* under present and future climate projections. Future models include years 2030, 2050, and 2080 under the 2.6, 4.5, 6.0, and 8.5 RCPs. The scale indicates less suitable environment (cooler colors) and most suitable environment (warmer colors).

Of the 80 individual models (8 algorithms * 10 evaluation runs) generated by BIOMOD2 for present environmental conditions, 17 models scored a TSS >0.6 (TSS_average_ = 0.63; Kappa_average_ = 0.58, ROC_average_ = 0.88). These 17 models incorporated weighted runs from GBM, GAM, CTA, and RF algorithms to create a final ensemble model. In this ensemble model, annual precipitation had the highest overall contribution (17.2%), followed by annual mean temperature (16.9%), elevation (16.1%), precipitation of the warmest quarter (14.1%), minimum temperature of the coldest month (13.4%), isothermality (11.9%), and mean temperature of the coldest quarter (10.5%). Under present climate conditions, the ensemble model indicated high environmental suitability for *Bd* throughout the Cameroon Volcanic Line, along the coast reaching into Equatorial Guinea and Nigeria, and towards central Cameroon including Mbam Djerem National Park (Fig 2). There are also pockets of high environmental suitability in other forested regions, such as in Lobéké National Park in the southeast and in Campo Ma’an National Park in the southwest of the country.

Fig 2 shows the projection models under future climate scenarios. Projection for RCP 2.6, which assumes that global annual greenhouse gas emissions peak between 2010–2020 and decline substantially thereafter, shows slight decreases in the range of highly suitable environment for the montane region and central Cameroon, but increases in the southern regions surrounding Campo Ma’an and Lobéké National Parks from years 2030 to 2080. Projection for RCP 4.5, which assumes emissions peak at 2040 and then decline, predicts similar patterns for the montane region and areas around Campo Ma’an. However, the Mbam Djerem region is predicted to decrease in its degree of environmental suitability but increase in area, while the Lobéké region is likely to see little change. Projection for RCP 6.0, which assumes emissions peak around 2080 and then decline, again predicts an increase in suitability for the Campo Ma’an region as well as a slight reduction in highly suitable area around the highlands over time. It also shows patchy changes in high environmental suitability in the Mbam Djerem region. The projection for RCP 8.0, which assumes emissions continue to rise, shows a significant reduction in suitable area in the northeastern region of the highlands for year 2030. However, this is followed by an increase in environmental suitability in this region as well as in the area near Campo Ma’an National Park by 2050. In year 2080, suitable area around Campo Ma’an decreases dramatically, while areas in northern Cameroon increase in environmental suitability. Notably, all future projection models predict high environmental suitability for *Bd* in areas surrounding the highland region.

### Phylogenetic analysis

**Fig 3:**
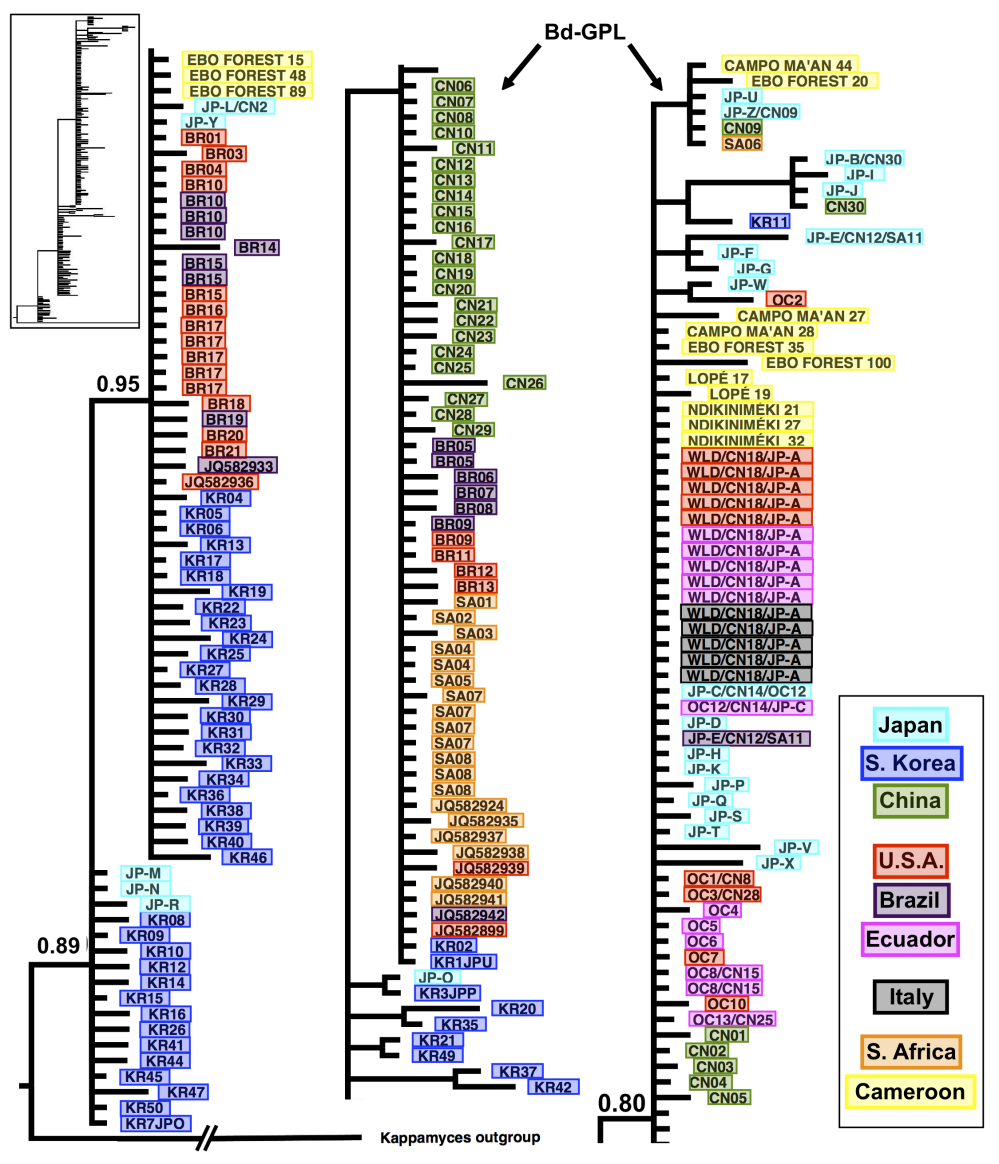
Phylogenetic tree of ITS rRNA sequences from global *Bd* samples. Isolates are color coded according to the country where they were collected. Samples from Cameroon are highlighted in yellow.

The phylogenetic tree of ITS rRNA haplotypes from worldwide *Bd* samples recovered similar relationships as reported in previous studies [71]. Basal haplogroups are dominated by haplotypes from South Korea and Brazil. Most of the Cameroon samples (n = 11) group with the *Bd*-GPL lineage whereas three fall within the basal clade (Fig 3 & S1 Fig). Samples from Campo Ma’an, Ebo Forest, Lobéké, and Ndikiniméki were identified as *Bd*-GPL, while additional samples from Ebo Forest cluster with the more basal clade. The *Bd*-GPL clade has high posterior probability (PP = 0.80) and contains haplotypes originating from Japan, China, South Africa, Italy, United States, and Ecuador.

## Discussion

### Spatial distribution, prevalence and infection intensity of *Bd* and its host associations

Considering the threat *Bd* poses to amphibians, it is essential to better understand potential patterns and drivers of distribution and prevalence. This requires surveying unsampled areas as well as monitoring previously sampled areas whenever possible. The overall low prevalence across the surveyed sites in Cameroon is consistent with recent assessments for Cameroon and other African regions. Surveys from 11 sites in Cameroon between 2007 and 2011 found an average *Bd* prevalence of 10.9%, whereas the average prevalence in the present study was 14.4% [17]. However, *Bd* had not been detected in Campo Ma’an [17], while the current study detected *Bd* at this site with a prevalence of 15.6%. *Bd* prevalence is associated with strong seasonal variation in temperature [72,73] and precipitation regimes [74]. Thus, it is possible that *Bd* prevalence is under-estimated in this study because samples were collected between the end of the dry season and beginning of the rainy season in Cameroon (March–July), when conditions are less suitable for infection.

*Bd* has been detected consistently at high elevations in Cameroon and neighboring regions. Here, *Bd* was detected at 23.5% prevalence at the higher elevation site on Mt. Cameroon. This is similar to the levels of prevalence estimated from more extensive field work on Mt. Oku and Mt. Manengouba in Cameroon [23] as well as in the Albertine Rift [75]. However, only one species (*Wolterstorffina parvipalmata*) was found at comparatively high abundance. This semi-arboreal species lays its eggs in water filled tree holes [76]. This behavior could facilitate *Bd* persistence in an area with low amphibian host diversity.

### Species distribution modeling

Areas of environmental suitability observed from the SDMs are those with increased annual precipitation, overall warmer temperatures, and at higher elevation. These environmental conditions are found in several regions of Cameroon and are highly suitable for *Bd*, causing potential concern for the diverse amphibian communities inhabiting these regions [77]. Under most climate change projections, Campo Ma’an, Mbam Djerem, and Lobéké National Parks are predicted to retain areas with high environmental suitability. Prevalence is currently relatively low for these areas and amphibian declines have not been reported, making the apparent risk of *Bd* outbreaks low. However, considering these are protected areas, we should pay close attention to amphibian communities in light of the current *Bd* presence and the future changing environments.

The Cameroon highlands, a hotspot of African amphibian diversity and a region experiencing amphibian declines, consistently make up a large proportion of suitable environment for *Bd*. Further, our models of future climate scenarios suggest that high environmental suitability in the montane regions is likely to persist into the future, likely driven by increasing average annual temperatures. For montane frogs, research suggests that the incidence of disease depends heavily on the effect of temperature on the host’s resistance to chytridiomycosis, rather than an effect of the fungus alone [78]. Many amphibians have narrow elevational ranges [79], and may become more vulnerable to disease outbreak due to shifts in temperature and precipitation that are favorable to the pathogen. This effect may be further compounded by the simultaneously decrease in available habitat for its montane amphibian hosts. Further work is needed to increase knowledge of amphibian communities and their distributions along elevational gradients in Cameroon to better understand patterns of infection at different elevations.

The SDMs presented here predict that Cameroon is likely to experience considerable shifts in the overall area and degree of environmental suitability for *Bd* under future climate conditions. While the climate-linked epidemic hypothesis has not been supported in areas of Central and South America [80], climate change has been linked to infection susceptibility in other areas where *Bd* has been introduced and causing amphibian declines, such as western Europe[81]. Environmental factors may be more reliable predictors of *Bd* in areas where is not alien and thus can help predict environmental suitability. In addition to understanding environmental factors limiting *Bd*’s distribution, future research in this region should explore host-related factors, such as variation in infection susceptibility, that may influence *Bd*’s success in an area and incorporate those factors into predictive models.

### Phylogenetic analysis

Molecular analyses show that the hypervirulent *Bd*-GPL was found at four of the eight sampling sites. These are all lower elevation forested sites in the center, south and east of the country. Two of these sites where the hypervirulent lineage is found (Ebo Forest and Ndikiniméki) could potentially be linked to populations declines in montane areas reported by Hirschfeld et al [23]. On the other hand, since *Bd*-GPL is considered to have arrived prior to recent amphibian population declines [35], amphibians in this region might offer insight into mechanisms of *Bd* tolerance or infection avoidance if current declines are not the result of *Bd*-GPL.

Other *Bd* samples in this study cluster with the lineages from Brazil and South Korea. While neither Brazilian nor South Korean haplotypes have been associated with amphibian declines [71], understanding how these haplotypes spread to or from Cameroon could offer insight into *Bd* dispersal. The presence of these global lineages could also be explained by the Cameroon wildlife and pet trade. Historical trade in native *Xenopus laevis* has been suggested as potential mechanism for the global spread of *Bd* [82]. *Bd* has also been detected in African caecilians from Cameroon involved in the amphibian pet trade [8] and infected Cameroon consignments of amphibians are considered very likely to have introduced of *Bd* into the United Kingdom [83]. Considering *Bd* is extremely resilient and can grow on a variety of potential carriers, such as dead amphibian and snake skin, dead algae, insect exoskeletons and feathers [84], its transmission from and into wild populations through trade or scientific facilities is not unlikely. It is also possible that the similarities observed between haplotypes from Cameroon, Brazil and South Korea are due to the retention of shared ancestral variation that has not been revealed due to the lack of sampling from many areas across the world.

Although existing primers have been shown to be highly specific to the *Bd* ITS1-5.8S-ITS2 region [63,64,71,85,86], the derived phylogenies assembled with the ITS region should be observed with caution because of this region’s high copy number, short length and lack of phylogenetic informativeness [86]. Future work should subject samples from Central Africa to a more rigorous genome-wide sequencing to better define the phylogenetic relationships of global samples.

## Conclusion

While there is still much to be discovered regarding *Bd* diversity and host-pathogen-environment interactions, the present study provides further insight into genetic diversity and environmental suitability of *Bd* in an important amphibian biodiversity hotspot in Equatorial Africa. The Cameroon highlands, and specifically Mt. Cameroon, are designated as high conservation priority areas [87]. Presence of the hypervirulent *Bd*-GPL in this region, while not detected at the montane site, could be potentially linked to the community declines observed in the highlands. With ongoing habitat destruction and degradation, as well as reductions of species climate envelope, amphibians could be pushed into smaller pockets of habitat that could potentially exacerbate *Bd* transmission due to over-crowding. Much progress has been made monitoring amphibian populations in the light of *Bd* emergence, but more work is needed to fully understand the environmental drivers of habitat suitability and map more precisely the global distribution of *Bd* lineages. To further understand the level of threat that *Bd* poses to Central African amphibians it is important for future research to further characterize *Bd* lineages in this region and determine how susceptible local amphibians are to different strains. Infectious diseases, primarily chytridiomycosis, are considered a major threat to amphibian populations so understanding which factors contribute to or limit infection across the globe is essential for amphibian conservation.

## Supporting information

S1 Table Species sampled at eight sites in Cameroon. (XLSX)

S2 Table Current and supplementary *Bd* occurrence locations used in BIOMOD2 analyses. (XLSX)

S3 Table Additional *Bd* sequences used in phylogenetic analysis. (XLSX)

S4 Table Infection intensities of *Bd* positive samples from Cameroon. (XLSX)

S5 Table *Bd* prevalence within amphibian groups from eight sites in Cameroon. (XLSX)

S1 Fig ITS phylogenetic tree. (TIFF)
